# Complex domain interactions regulate stability and activity of closely related proneural transcription factors

**DOI:** 10.1016/j.bbrc.2014.06.127

**Published:** 2014-08-08

**Authors:** Gary S. McDowell, Laura J.A. Hardwick, Anna Philpott

**Affiliations:** Department of Oncology, University of Cambridge, Hutchison/MRC Research Centre, Cambridge Biomedical Campus, Cambridge CB2 0XZ, UK

**Keywords:** bHLH, basic Helix-Loop-Helix, Ngn2, Neurogenin 2, UPS, Ubiquitin–Proteasome System, Ub, ubiquitin, ANOVA, analysis of variance, UD, unfolded domain, *Xenopus*, Ubiquitylation, Neurogenesis, Proneural, Proteasomal degradation

## Abstract

Characterising post-translational regulation of key transcriptional activators is crucial for understanding how cell division and differentiation are coordinated in developing organisms and cycling cells. One important mode of protein post-translational control is by regulation of half-life via ubiquitin-mediated proteolysis. Two key basic Helix-Loop-Helix transcription factors, Neurogenin 2 (Ngn2) and NeuroD, play central roles in development of the central nervous system but despite their homology, Ngn2 is a highly unstable protein whilst NeuroD is, by comparison, very stable. The basis for and the consequences of the difference in stability of these two structurally and functionally related proteins has not been explored. Here we see that ubiquitylation alone does not determine Ngn2 or NeuroD stability. By making chimeric proteins, we see that the N-terminus of NeuroD in particular has a stabilising effect, whilst despite their high levels of homology, the most conserved bHLH domains of these proneural proteins alone can confer significant changes in protein stability. Despite widely differing stabilities of Ngn2, NeuroD and the chimeric proteins composed of domains of both, there is little correlation between protein half-life and ability to drive neuronal differentiation. Therefore, we conclude that despite significant homology between Ngn2 and NeuroD, the regulation of their stability differs markedly and moreover, stability/instability of the proteins is not a direct correlate of their activity.

## Highlights

• Although closely related, Ngn2 is rapidly degraded whereas NeuroD is stable.
• NeuroD is ubiquitylated but not degraded.
• The N-terminal domain of NeuroD confers stability.
• Conserved bHLHs of Ngn2 and NeuroD promote instability/stability respectively.
• Stability of chimeric proteins is not correlated with differentiation activity.

## 1 Introduction

Basic Helix-Loop-Helix (bHLH) transcription factors play a central role in cell fate and differentiation in a wide variety of tissues, often by acting as master regulators coordinating expression of multiple downstream targets [1]. Tissue-specific class II bHLH proteins contain a DNA-binding basic domain, followed by two α-helices separated by a loop, and flanked either side by regions of poorly defined structure [2]. Structure and function studies have shown that these transcriptional regulators act as heterodimers with the ubiquitously expressed class I bHLH E2A gene products E12 or E47; the Helix-Loop-Helix (HLH) domain mediates heterodimerisation whilst the basic region binds to a consensus E-box DNA motif in the promoter region of target genes [3], [4].

One member of this family, Neurogenin 2 (Ngn2), acts as a master regulator of neurogenesis in regions of the central nervous system [5]. Ngn2 is essential for neuronal differentiation during primary neurogenesis in the *Xenopus* frog embryo [6] and induction of ectopic neurons in *Xenopus* by Ngn2 has been widely used to study Ngn2 function [7], [8], [9]. Differentiation of these primary neurons also absolutely requires activity of an additional related bHLH transcription factor, NeuroD [10]. In *Xenopus*, it has been shown that Ngn2 both upregulates NeuroD expression in a unidirectional cascade, and functions in parallel with NeuroD, activating a large number of common target genes required for primary neurogenesis [11]. Yet even with their structural and functional similarities, the half-life of these proteins differs significantly [12]. The basis for this difference and its functional consequences have not been investigated.

Transcription factors tend to be highly unstable proteins degraded by the Ubiquitin–Proteasome System (UPS) [13]. To target proteins for destruction, Ubiquitin (Ub) is activated and covalently fused to a specific substrate protein at electron-rich sites (usually lysines, reviewed in [14]). Ubiquitylation can be repeated to build up a chain of at least 4 Ub moieties that then targets the substrate to the 26S proteasome [15]. Using energy from ATP hydrolysis, ubiquitylated proteins are then unfolded from an unfolding initiation site [16] and cleaved into small peptides. This regulation generally results in highly dynamic protein levels, which adjust in response to intrinsic and extrinsic controls.

We have previously shown that Ngn2 is rapidly degraded by the UPS, whereas NeuroD is stable under similar conditions [12]. Unusually, this rapid degradation of Ngn2 is brought about by both canonical ubiquitylation on lysine residues, and non-canonical ubiquitylation on cysteines, serines and threonines [17], [18]. The structural aspects of NeuroD and Ngn2 that confer stability/instability have not been explored, and whether differences in stability relate to differences in ubiquitylation, or whether they relate to differences in destruction of ubiquitylated proteins is yet to be determined. Moreover, the relationship between proneural protein half-life and ability to activate downstream target activation and drive neurogenesis remains unknown.

In this study we compare the roles of protein structure and ubiquitylation in regulating Ngn2 and NeuroD stability and activity by undertaking a domain-swap analysis between the two proteins. We show that similarly structured proteins do not necessarily exhibit similar biochemical properties with respect to ubiquitylation and degradation. Furthermore, we show that there is poor correlation between protein half-life and protein activity *in vivo*.

## 2 Materials and methods

### 2.1 Cloning

Point-mutant constructs were made by site-directed mutagenesis (Stratagene) and cloned into pCS2+ as described previously [12], [17] using standard methods.

#### 2.1.1 Unfolded domains

Unfolded domain constructs were a kind gift of Andreas Matouschek [19]. The domains were fused to the N- and C-termini of NeuroD using the Gateway® cloning system (Invitrogen). **NeuroD-UD**: NeuroD DNA was amplified by PCR between attB1 and att5Br sites: Forward ATGACCAAATCGTATGGAGAGAATGG, Reverse TTAATCATGAAAGATGGCATTTAGCTGG. UD DNA was amplified between attB5 and attB2 sites: Forward ATGCTAAAATACAAACCTTTAC, Reverse TTATTCAGCGGGCGAAAATC. **UD-NeuroD**: NeuroD DNA was amplified by PCR between attB5 and attB2 sites: Forward ATGACCAAATCGTATGGAGAG, Reverse ATCATGAAAGATGGCATTTAGC. UD DNA was amplified between attB1 and att5Br sites: Forward ATGCTAAAATACAAACCTTTAC, Reverse TTCAGCGGGCGAAAATCTTTTG.

#### 2.1.2 Domain-swaps

Domain-swapped mutants were produced using primers containing Ngn2 fused to NeuroD sequence, so that there was no artificial linker between the domains of the proteins. The PCR products of the N-terminal portion of the domain-swap were used as the forward primers in a second PCR reaction, using a plasmid encoding the other protein as the vector. The primers at the extreme N- and C-termini of the final domain-swapped product lie between BamHI and XhoI restriction sites, with a Kozak sequence before the initiation site.

#### 2.1.3 Primer sequences (where primers overlap, the Ngn2 sequence is in bold)

**N-Ngn/BC-NeuroD**, Ngn2 portion: Forward: ATGGTGCTGCTCAAGTG, Reverse: **TAAAGATCAAGAAGACC**AGACGCATGAAGGCAAA; N-Ngn/BC-NeuroD full protein: Forward: Ngn2 portion, Reverse: TTAATCATGAAAGAT.

**NB-Ngn/C-NeuroD**, Ngn2 portion: Forward: ATGGTGCTGCTCAAGTG, Reverse: **TTAGCGAAACTTTGCGC**TCCGGCAAAAGCCCAGA; NB-Ngn/C-NeuroD full protein: Forward: Ngn2 portion, Reverse: TTAATCATGAAAGAT.

**N-Ngn/BC-NeuroD** full protein: Forward: Ngn2 portion, Reverse: TTAATCATGAAAGAT. N-NeuroD/BC-Ngn, NeuroD portion: Forward: ATGACCAAATCGTATGGA, Reverse: TGGAGCGATTTAAAGTG**CGGCGCGTTAAAGCTAA**; N-NeuroD/BC-Ngn full protein: Forward: NeuroD portion, Reverse: TCAAATGAAAGCGCT.

**NB-NeuroD/C-Ngn**, NeuroD portion: Forward: ATGACCAAATCGTATGGA, Reverse: TTTCTGAGATTTTAAGG**CTTGGCGACCCAGTGCA**; NB-NeuroD/C-Ngn full protein: Forward: NeuroD portion, Reverse: TCAAATGAAAGCGCT.

For NgnNDNgn and NDNgnND proteins, the domain-swapped plasmids above were used as vectors for the PCR reaction of the C-terminal portion of the protein e.g. for NgnNDNgn the N-terminal Ngn2 PCR product (Forward: ATGGTGCTGCTCAAGTG, Reverse: **TAAAGATCAAGAAGACC**AGACGCATGAAGGCAAA) was used as the forward primer and the reverse primer was TTAATCATGAAAGAT, using NB-NeuroD/C-Ngn as the vector.

### 2.2 *In Vitro* Translation

TNT® SP6 quick coupled transcription/translation system (Promega), with ^35^S-methionine (GE Healthcare), was carried out according to the manufacturer’s instructions.

### 2.3 *Xenopus* extracts

Activated interphase egg extracts [12], mitotic egg extracts [17] and neurula embryo extracts [18] were prepared as described previously.

### 2.4 Degradation assays

Degradation assays were performed as described previously [17].

### 2.5 Ubiquitylation assays

Ubiquitylation assays were performed as described previously [18].

### 2.6 Clustal W2 analysis

Clustal W2 analysis was carried out to align protein sequences [20].

### 2.7 *Xenopus laevis* embryos

Acquisition of *Xenopus laevis* embryos, preparation and injection of synthetic mRNA, staging of embryos and *in situ* hybridisation and qPCR were conducted as described previously [7], [21].

### 2.8 Multiple comparison testing

Multiple comparison tests were carried out on the log_2_-transformed ratios of protein half-lives compared to wild type. Analysis was carried out with MATLAB® by one-way analysis of variance (ANOVA) followed by a multiple comparison test using the statistical output of the ANOVA. Statistical significance of the differences between the means was determined using a critical level of alpha of 0.05.

## 3 Results and discussion

### 3.1 Both Ngn2 and NeuroD are ubiquitylated but only Ngn2 is degraded

**Fig. 1:**
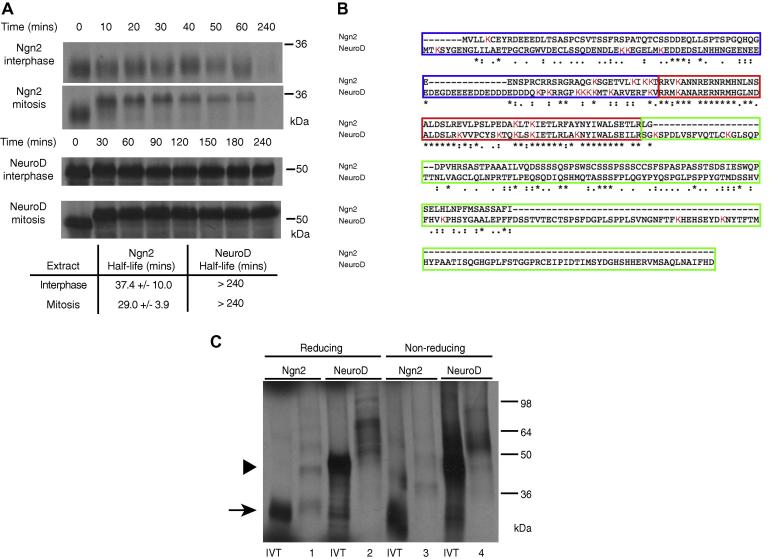
Ngn2 is degraded whilst NeuroD is stable despite being ubiquitylated. (A) *X. laevis* interphase and mitotic egg extracts were supplemented with IVT ^35^S-labelled Ngn2 or NeuroD and incubated at 21 °C. Samples at increasing time points were analysed by SDS–PAGE followed by autoradiography and quantitative phosphorimaging analysis, calculating the half-lives using first-order rate kinetics, and errors calculated using the Standard Error of the Mean (SEM). *n* = 2. (B) ClustalW2 [20] analysis of sequences from Ngn2 and NeuroD. The N-terminal domain is bordered in blue; the bHLH domain in red; and the C-terminal domain in green. Lysine residues are highlighted in red. (C) Interphase egg extracts were supplemented with IVT ^35^S-labelled Ngn2 or NeuroD in the presence of MG132 and His_6_-ubiquitin and incubated at 20 °C for 90 min. Samples were bound to Ni–NTA beads and subjected to SDS–PAGE in reducing or non-reducing conditions and analysed by autoradiography. Lanes are numbered 1–4 as described in the text. *n* = 2. (For interpretation of the references to colour in this figure legend, the reader is referred to the web version of this article.)

*Xenopus* egg extracts contain all necessary components of the ubiquitin–proteasome machinery for *in vitro* study of protein degradation. We have previously reported that Ngn2 protein is degraded rapidly in interphase *Xenopus* egg extract [7], whereas NeuroD is stable [12]. Given that Ngn2 is less stable in mitosis than interphase [17], we determined whether NeuroD degradation was enhanced in mitosis. Degradation assays were performed *in vitro* using *Xenopus* egg extracts, comparing degradation rates of Ngn2 and NeuroD during both interphase and mitosis. Whilst Ngn2 was indeed more unstable in mitotic compared to interphase extract, NeuroD was stable in both (Fig. 1A).

Ngn2 is ubiquitylated on canonical lysine residues, and additional non-canonical sites such as the N-terminus and serine/threonine/cysteine residues [17], [18]. Whilst NeuroD contains more potential canonical ubiquitylation sites (lysines) than Ngn2, particularly in the conserved bHLH domain (highlighted in Fig. 1B), stability of NeuroD could result from a lack of ubiquitylation on these sites. Hence, we next investigated whether radiolabelled NeuroD would undergo *in vitro* ubiquitylation in *Xenopus* egg extract. Despite the difference in their stability, both Ngn2 and NeuroD were ubiquitylated in *Xenopus* extracts, as evidenced by ladders of poly-ubiquitylated proteins on SDS–PAGE after his-Ub pulldown on NTA-agarose beads [17] (Fig. 1C, lanes 1–4). Therefore ubiquitylation alone does not explain the difference in degradation rates between the two proteins.

Non-canonical ubiquitylation of Ngn2 can occur on cysteine residues [17], [18] via disulphide bonds [14]. When pulling down poly-ubiquitylated proteins, any Ngn2 linked to his-Ub chains via cysteine linkages will be released under the reducing, high pH conditions [17], [18] to run as unconjugated protein on SDS–PAGE. As expected, unconjugated Ngn2 protein was released in high pH/reducing conditions (compare Fig. 1C, lanes 1 and 3, arrow), confirming ubiquitylation on non-canonical sites ([17], [18], reviewed in [14]). However, unconjugated NeuroD is not released by high pH/reducing conditions (Fig. 1C, lane 2, arrowhead). Therefore whilst both proteins were ubiquitylated, non-canonical residues such as cysteines are targeted only on Ngn2 and not on NeuroD. However, as Ngn2 is still efficiently targeted for degradation even in the absence of cysteine ubiquitylation [18], this also cannot solely account for the stability difference between the two proteins.

### 3.2 NeuroD is not destabilised by addition of an unfolding initiation site

**Fig. 2:**
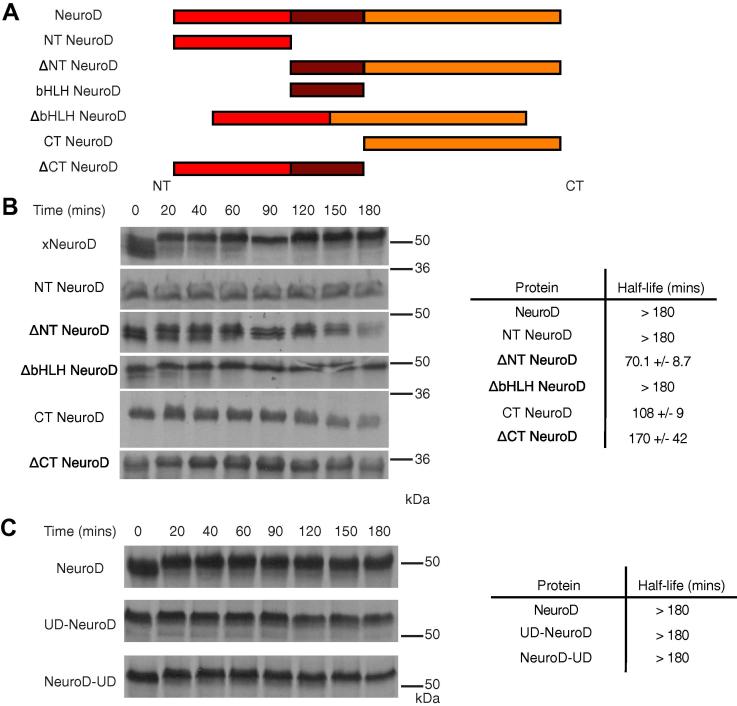
Stability of NeuroD domains and fusions between NeuroD and an unfolding domain. (A) Schematic of NeuroD domain deletion mutants. (B) NeuroD domain deletion mutants were subjected to degradation assay in interphase egg extract. *n* = 3. (C) Unfolding domain-fused (UD) proteins were subjected to degradation assay in interphase egg extract. *n* = 3.

For degradation to occur, an unfolding initiation site is required in addition to polyubiquitylation and regions resistant to unfolding may impede ubiquitin-mediated destruction [16]. To determine whether NeuroD stability is influenced by inappropriate ubiquitin linkages or structural constraints against degradation, we expressed different domains of NeuroD and assayed their relative stability in interphase egg extract. The small bHLH domain of NeuroD could not be expressed in reticulocyte lysate, indicating an inherent instability that precluded further study of this domain in isolation. Instead we investigated NeuroD truncation mutants of the N and C-termini, with and without the bHLH domain (see Fig. 2A for schematic).

Deletion mutants that contained the C-terminus of NeuroD, either with or without the bHLH domain, showed significantly reduced protein half-life compared to full-length NeuroD. In contrast, all constructs containing the N-terminus of NeuroD, with or without the bHLH domain, had a substantially greater half-life (Fig. 2B). One possibility for the enhanced stability of the N-terminus of NeuroD is that the N-terminal domain does not provide the unstructured region required to initiate proteasomal unfolding, or alternatively, the N-terminal domain could actively impede NeuroD degradation. To distinguish between these possibilities, we fused an unfolded domain (UD) onto the N- or C-terminus of NeuroD. This UD consisted of residues 1–95 of the mitochondrial precursor protein cytochrome b_2_ [19] that promotes unfolding of the heterologous proteins to which it is fused. The stabilities of these UD-fused NeuroD proteins were then compared with wild-type NeuroD in interphase *Xenopus* extract (Fig. 2C). Neither N- nor C-terminal fusion of the UD to NeuroD reduced protein half-life, indicating that adding an unfolding domain was not sufficient to bring about destabilisation.

### 3.3 Ngn2 and NeuroD domain-swapping

**Fig. 3:**
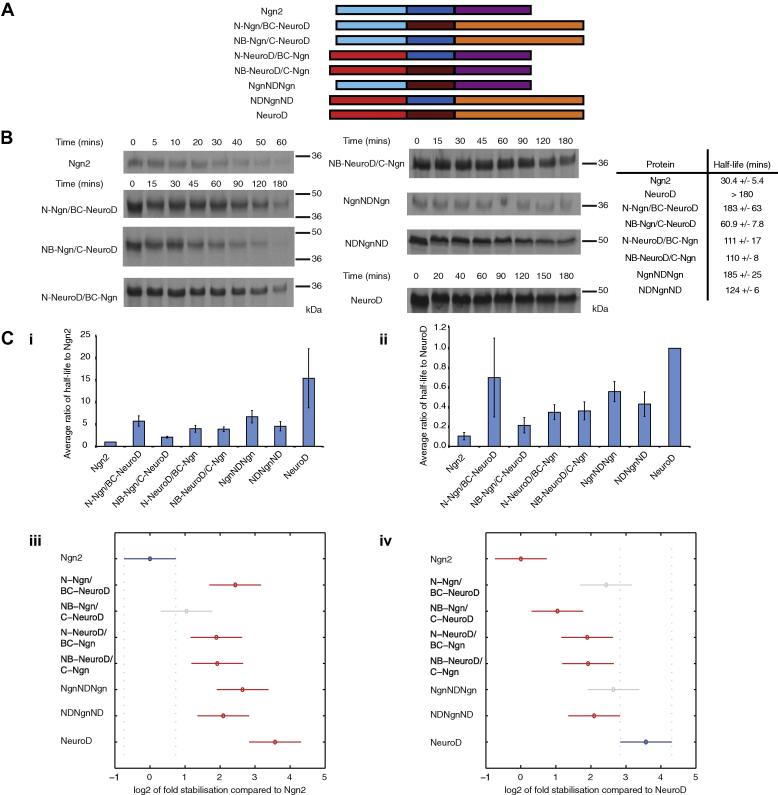
Analysis of stability of domain-swapped mutants of Ngn2 and NeuroD. (A) Schematic representation of Ngn2 and NeuroD domain-swap proteins. (B) Domain-swapped proteins were subjected to degradation assay in interphase egg extract. *n* = 4. (C) The average stabilisation relative to wild type (i) Ngn2 and (ii) NeuroD was calculated. Analysis of variance (ANOVA) was performed to determine which proteins were significantly different from (iii) Ngn2 and (iv) NeuroD and these are shown as red bars (reference proteins denoted by blue bars; grey bars show no significant difference from wild type). (For interpretation of the references to colour in this figure legend, the reader is referred to the web version of this article.)

Having established that Ngn2 is unstable whilst NeuroD is stable, and demonstrating a role for the N-terminus of NeuroD in contributing to its stability, we next examined the relationship between protein half-life and domain identity using a further series of domain-swapped constructs. We generated mutants of Ngn2 and NeuroD, in which the N-and C-termini, with and without the bHLH domain, were swapped between the two proteins; stability of the hybrid proteins was then determined as described previously (Fig. 3).

When comparing the half-life of the fusion proteins to that of Ngn2, substitution of any domain of Ngn2 for the corresponding domain of NeuroD resulted in a stabilisation compared to wild type Ngn2. Substituting the N-terminus of NeuroD into Ngn2 resulted in at least a 3-fold increase in protein stability. Furthermore, consistent with the greater stability of the N-terminal domain of NeuroD alone, substituting the C-terminus of NeuroD into Ngn2 had a relatively smaller effect on increasing stability than the N-terminal substitution (Fig. 2B, C).

Perhaps surprisingly, even though the bHLH domains of the two proteins are 70% identical at the amino acid level, replacing the bHLH domain of Ngn2 with that of NeuroD resulted in 4-fold stabilisation compared to wild type Ngn2. Conversely, substituting the NeuroD bHLH with that of Ngn2 led to a protein with almost half the stability of the wild type NeuroD (Fig. 3B, C). Hence, the bHLH domain also plays an important role in determining the half-life of both NeuroD and Ngn2. However, it is clear that there is strong interplay between domains in determining half-life and the bHLH domain is not the sole determinant of protein stability as the N-terminus and bHLH domain of NeuroD fused to the C-terminus of Ngn2 has a shorter half-life (110 min ± 8) than the fusion of N-and C-termini of Ngn2 either side of the NeuroD bHLH (185 min ± 25).

Taken together, Ngn2 and NeuroD have markedly differing half–lives in *Xenopus*, even though they show strong homology and functional overlap. Both proteins are ubiquitylated, although Ngn2 shows non-canonical ubiquitylation on cysteines that is not observed in NeuroD. Truncation mutants demonstrate that whilst the C-terminus of NeuroD can be degraded, the N-terminus confers stability with or without the bHLH domain. When domain-swap mutants are made between Ngn2 and NeuroD, the bHLH domain of Ngn2 is destabilising, whilst all domains of NeuroD are stabilising. Nevertheless, we can conclude that stability/instability is not conferred by any single domain of either protein, but is protein context-dependent. Hence, final protein half-life must be a result of interaction between ubiquitylation and intrinsic stability of all domains of the protein. This is in contrast to proteins such as p42 protein in Influenza C virus, where stability is regulated by one part of the protein and can be transferred to effect the degradation of another protein [22].

Upon overexpression in *Xenopus* embryos, both Ngn2 and NeuroD can induce ectopic neurogenesis and the proteins share many common downstream targets [11]. DNA binding resides in the basic region, which is 85% homologous at the amino acid level between the two proteins, and heterodimerisation to their common E-protein partners occurs via the HLH domain [5]. There are likely to also be important, though ill-defined, interactions with the N- and C-terminal domains that will affect both target specificity and transcriptional activity, possibly through cofactor/regulator binding. The impact of protein half-life on the ability to drive neurogenesis has not been explored. One might expect a stabilised protein to have greater transcriptional activity and so be more efficient at driving neuronal differentiation. However, studies have also suggested that intrinsic instability might be a requirement for transcriptional activation [23], [24]*.* Hence, we investigated the relationship between the half-life of the domain-swapped fusion proteins and their ability to drive neurogenesis.

**Fig. 4:**
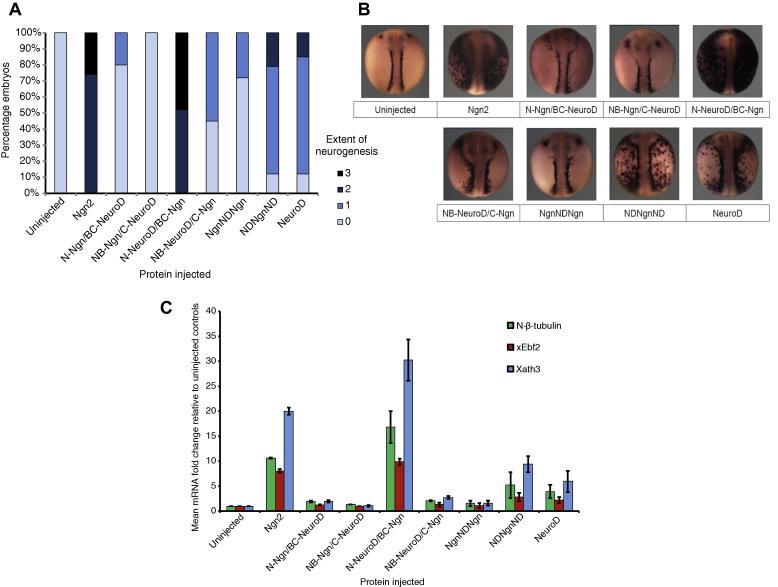
*In vivo* proneural activity assayed in developing *Xenopus* embryos. *X. laevis* embryos were injected at one cell stage with 50 pg of mRNA encoding chimeric constructs as indicated. Embryos were fixed at Stage 19 for ISH analysis of neural β-tubulin expression and scored for the extent of neurogenesis relative to uninjected controls. (A) Semi-quantitative ISH scoring data [*n* = 22–36], 0–3 where 0 indicates no increase in neurogenesis to 3, highly extensive neurogenesis throughout the epidermis. (B) Representative images of embryos injected with each construct. (C) *X. laevis* embryos were injected at one cell stage with 100 pg domain-swap mRNA and analysed by qPCR at stage 19 for neural β-tubulin, xEbf2 or Xath3 expression, relative to uninjected embryos. *n* = 2. Errors are Standard Error of the Mean (SEM).

To assess whether stability tracks with activity, mRNA coding the chimeric proteins was injected into fertilised one-cell *Xenopus* embryos. The extent of neurogenesis was assayed in Stage 19 embryos by *in situ* hybridisation (ISH) to detect neural β-tubulin (Fig. 4A, B), allowing a semi-quantitative comparison of activity between proteins. For a more quantitative readout of relative activity, qPCR assays were performed to measure expression of neural β-tubulin, the marker of neuronal differentiation, and xEbf2 and Xath3 (Fig. 4C), downstream targets common to both Ngn2 and NeuroD [11]. Both assays gave similar results with respect to chimeric protein activity.

Overexpression of both Ngn2 and NeuroD resulted in ectopic neurogenesis, with Ngn2 the more potent of the two molecules. Although both proteins have some overlapping targets that promote neurogenesis, Ngn2 acts as a master regulator of primary neurogenesis whilst NeuroD acts downstream of Ngn2 in cells that have already committed to neuronal differentiation [6], [9], [11].

When comparing the activities of chimeric proteins, the importance of the bHLH domain for driving neuronal differentiation is evident, and simply substituting the bHLH of Ngn2 with that of NeuroD renders the chimera NgnNDNgn inactive. However, making the reciprocal swap of the Ngn2 bHLH into NeuroD has little effect on NDNgnND activity when compared to the activity of wild type NeuroD (Fig. 4A). Most chimeric proteins show significantly reduced activity compared to wild type Ngn2 with the striking exception of N-NeuroD/BC-Ngn, the chimera containing the N-terminal domain of NeuroD fused to the bHLH and C-terminal domain of Ngn2, which showed activity similar to, if not greater than, Ngn2.

Therefore, the bHLH domain is important for regulating neurogenic activity, yet there are additional interactions involving both the N- and C-terminal domains that can influence activity. These may include intramolecular interactions between the domains of the protein molecule, or intermolecular interactions, either within the heterodimeric bHLH/E-protein complex, or more extensive protein–protein interactions mediating the assembly of larger multimeric transcriptional complexes. However, we found no correlation between the stability of the chimeras and their proneural activity *in vivo*; for instance Ngn2 is much more active than NeuroD, despite having a much shorter half-life, and N-NeuroD/BC-Ngn has a half-life 3–4 times as long as wild type Ngn2, yet shows similar activity.

The bHLH domain of Ngn2 is necessary but not sufficient to retain high-level neurogenic activity and whilst the C-terminal domain of Ngn2 confers high activity to the corresponding chimeric constructs, the N-terminal domain does not. Conversely, substituting either the bHLH or C-terminus of NeuroD into a chimera reduces the activity of the construct when compared to wild type Ngn2. Interestingly, the chimeric protein consisting of the N- and C-terminal domains of Ngn2 with the bHLH of NeuroD results in no ectopic neuron induction. This indicates that simply binding to the NeuroD E-box consensus sequence via the basic region is not enough to generate proneural activity, when the bHLH domain is surrounded by Ngn2 transcriptional activation domains.

Taken together, these data demonstrate that neither half-life nor DNA binding alone play a defining role in controlling the neurogenic activity of these closely related but distinct proneural proteins, but instead complex and coordinate interactions between the N- and C-termini and the bHLH domains are crucial for regulation. The nature of these interactions remains to be determined although the small amount of evidence available indicates that the N- and C-termini may be natively unstructured [25]. In such cases, conformation may be acquired by DNA- and protein partner-binding and such interactions may account for domain-specific requirements.

Proteins from similar families are often assumed to have similar folding mechanisms [26], similar regulation of degradation [12], [17], [18], [27], and/or similar control of transcriptional activity. However, as we show here, the regulation of proteins within closely related families can differ substantially and extrapolation between family members is unwise. It will be important to now understand how differences in protein behaviour and activity contribute to their differing roles in neurogenic determination and differentiation [6].
